# Part-Aware Point Cloud Completion through Multi-Modal Part Segmentation

**DOI:** 10.3390/e25121588

**Published:** 2023-11-27

**Authors:** Fuyang Yu, Runze Tian, Xuanjun Wang, Xiaohui Liang

**Affiliations:** School of Computer Science and Engineering, Beihang University, Beijing 100191, China; yfy1996@buaa.edu.cn (F.Y.); trz2000@buaa.edu.cn (R.T.); wangxuanjun@buaa.edu.cn (X.W.)

**Keywords:** computer vision, point cloud, multi-modal, 3D shape completion

## Abstract

Point cloud completion aims to generate high-resolution point clouds using incomplete point clouds as input and is the foundational task for many 3D visual applications. However, most existing methods suffer from issues related to rough localized structures. In this paper, we attribute these problems to the lack of attention to local details in the global optimization methods used for the task. Thus, we propose a new model, called PA-NET, to guide the network to pay more attention to local structures. Specifically, we first use textual embedding to assist in training a robust point assignment network, enabling the transformation of global optimization into the co-optimization of local and global aspects. Then, we design a novel plug-in module using the assignment network and introduce a new loss function to guide the network’s attention towards local structures. Numerous experiments were conducted, and the quantitative results demonstrate that our method achieves novel performance on different datasets. Additionally, the visualization results show that our method efficiently resolves the issue of poor local structures in the generated point cloud.

## 1. Introduction

Raw point clouds captured by 3D scanners and depth cameras are often sparse and incomplete due to the devices’ low resolution [1]. Point cloud completion, aimed at generating complete shapes from incomplete point clouds, enhances the usability of the collected data for subsequent tasks. Consequently, this task has garnered significant attention among researchers. Initially, geometry-based methods [2,3] were employed to fill in missing areas of point clouds. However, these methods proved inadequate when dealing with extensive missing sections of point clouds.

Due to advancements in deep learning and the availability of large-scale point cloud datasets, deep learning-based methods for point cloud completion have gained significant traction among researchers. These techniques, such as those proposed by Han et al. [4] and Litany et al. [5], focus on capturing the overall distribution of an object’s point cloud and can be applied to various scenarios. One of the primary challenges in deep learning-based approaches is enhancing the accuracy of the completion results.

FoldingNet [6] addresses this challenge by employing a two-stage generation process, assuming that the 3D object lies on a 2D manifold. This approach gradually generates a high-quality point cloud. Another method, PF-Net [7], predicts multi-scale complete point clouds, which are fused to achieve more precise results in subsequent steps. These innovative techniques represent significant strides in improving the accuracy of point cloud completion results.

**Figure 1:**
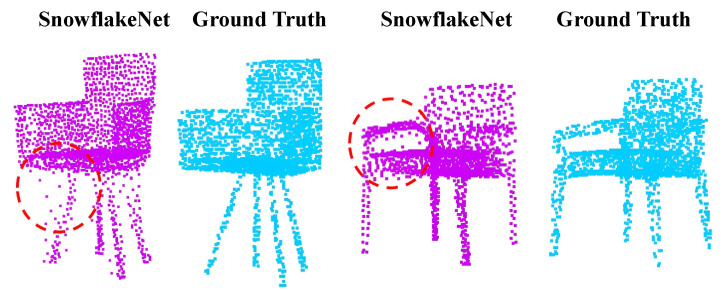
Despite Snowflake’s efforts to enhance part-completion effectiveness, it still struggles with inadequate local structures.

However, existing methods often struggle with preserving intricate local details within point clouds. Completing fine-grained elements like sharp edges and corners remains a challenging task. While certain techniques, like Snowflake [8], attempt to leverage hierarchical approaches to enhance local structures, they lack explicit guidance on how the network should refine these intricate details, also achieving bad local structures, as illustrated in Figure 1. The reason behind this limitation is their failure to effectively model the local structure, preventing them from optimizing local details in a specific and targeted manner.

**Figure 2:**
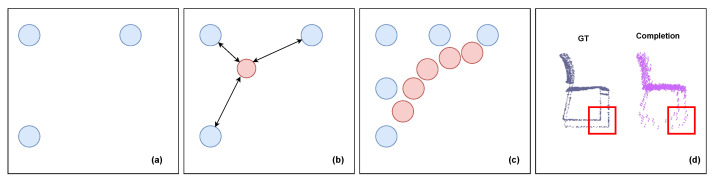
Ambiguity in structure arises from the use of the Chamfer Distance loss function. The blue circle represents the point cloud in the ground truth, while the red one represents the generated point cloud.

**Figure 3:**
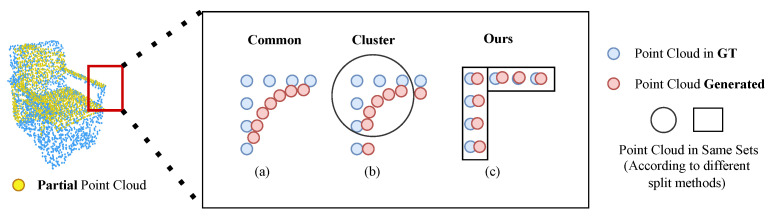
The analysis of the poor local structure in sharp regions is illustrated in the figures provided. For a sharp part, such as the armrest of the chair in the figure, the point cloud distribution of the ground truth is approximately presented at right angles, shown as blue points. As analyzed in Figure 2, in (**a**), it is evident that the generated point cloud tends to distribute on a smooth surface, leading to a local optimum. Figure (**b**) demonstrates that the straightforward clustering method fails to resolve the problem effectively. In contrast, in (**c**), our proposed splitting method successfully addresses the issue, providing an effective solution.

The problem we identify arises from the limitations of the commonly used optimization method, Chamfer Distance (CD). The loss function in use does not impose a one-to-one constraint. In Figure 2, the blue circle represents the ground-truth point cloud, while the red circle depicts the generated point cloud. Assuming that the ground truth forms a right triangle with its three points, illustrated in Figure 2a, the sum of the distances from the triangle vertices to the generated point is minimized when the blue point aligns with the triangle’s center of gravity, as seen in Figure 2b. This principle extends to Chamfer Distance, which minimizes the sum of the shortest distances between points in two sets of point clouds. With an expanding number of points, the resulting points tend to cluster within the structure, forming an arc, as seen in Figure 2c. At this juncture, inverse distance computation is inadequate for correction, leading the complementary result towards a local optimum, as depicted in Figure 2d. In essence, it tends to smooth sharp edges and corners, generating rounded surfaces. As depicted in Figure 3a, similarly, we choose a right-angled structure to investigate the solution. A straightforward solution involves dividing the overall point cloud into distinct sets. Although constraining the interaction between different sets can help the network escape local optima, a basic clustering method, as illustrated in Figure 3b, does not effectively address this issue. Sharp parts typically consist of numerous points with similar spatial coordinates, causing these points to be assigned to the same cluster. This situation introduces the same local optimum. Fortunately, we have observed that this problem can be resolved by dividing the point cloud into parts based on the overall direction of the local point cloud, as demonstrated in Figure 3c. The splitting method aligns closely with what is commonly known as semantic parts. Therefore, segmenting the point cloud according to semantic parts can effectively mitigate these challenges.

To address this problem, we must overcome three key challenges: (1) Mismatched granularity in datasets: The current datasets for point cloud part segmentation are not suitable for our segmentation needs because the granularity of their divisions does not align with our requirements. (2) Differences between generated and ground-truth point clouds: The generated point cloud might significantly differ from the ground truth. A robust splitting method is necessary to ensure that the parts segmented from these two point clouds are accurately aligned. (3) Need for suitable framework and constraints: A suitable framework, along with appropriate constraints, is essential to enabling the effective learning of this knowledge by the network.

To tackle these challenges, we introduce a novel framework, called PA-NET, which applies constraints directly to the segmented parts, guiding the network to generate intricate local details. Specifically, we introduce a robust multi-modal point cloud assignment module (MPCA) that allocates point clouds to different local parts. This method incorporates textual embeddings to address dataset limitations, ensuring a robust splitting process. We enforce semantic definitions on the output part set by aligning textual embeddings and part features in a specific order; thus, the module can split the coarse point cloud generated by the backbone network and the ground truth into aligned parts. Additionally, we propose a multi-stage part-aware refine module (MPR) for the co-optimization of parts and the global structure. This framework guides the network to learn local geometric features and produce results with clearly defined local structures. We introduce a novel loss function that measures part similarity guided by the assignment matrix given by the MPCA module. A refinement network is designed to transfer constraints to the features, directing the network’s focus towards local structures. To validate our method, we conducted extensive experiments, providing visualizations and analyses. The experimental results demonstrate that our approach achieves novel performance in accuracy and generates clearly localized structures.

Our primary contributions can be outlined as follows:

- We introduce a novel multi-modal part assignment module aimed at addressing the scarcity of suitable datasets, enabling the aligned part segmentation of coarse point cloud and ground truth.
- We introduce a plug-in module along with a corresponding part loss function designed to guide the network in learning local structures, enhancing local details.
- Extensive experiments and visualizations demonstrate that our method achieves novel performance, significantly enhancing localized structures.

## 2. Related Work

### 2.1. Deep Learning-Based Point Cloud Completion

PCN [9] was the pioneering work that introduced deep learning into point cloud completion. This approach utilized a network to model the mapping between residual and complete point clouds. Subsequent studies [10,11,12] have made significant contributions to this field. The recent focus has shifted towards obtaining more fine-grained results. These methods can be categorized based on different decoding approaches.

#### 2.1.1. Folding-Based Decoding

These methods operate under the assumption that 3D objects lie on a 2D manifold. The initial work, FoldingNet [6], employed a two-step network approach, utilizing 3D grid points to enhance result details. SA-Net [1] extended the generation process into multiple stages and introduced a hierarchical folding strategy for more precise outcomes. PU-GAN [13] incorporated Generative Adversarial Network (GAN) techniques to enhance robustness through the adversarial training of the encoder and decoder. ASHF-Net [14] introduced an adaptive sampling strategy, leading to a more robust method. While these techniques enhance point cloud surface quality, they do not explicitly model the local structure of the point cloud, making it challenging to generate results with improved local detail.

#### 2.1.2. Coarse-to-Fine Decoding

To enhance the accuracy of generated point clouds, a coarse-to-fine framework proves to be effective. These methods typically employ a two-stage approach, first generating a low-resolution point cloud and then refining it to higher resolution. This framework is highly interpretable and easy to control. PCN [9] and NSFA [15] were the pioneering works that introduced this framework to point cloud completion. Building upon this foundation, CDN [16] and PF-NET [7] added more generation stages to achieve superior results. More recently, Snowflake [8] introduced a point-wise splitting operation to enhance the decoder’s capabilities, achieving state-of-the-art performance. Despite the progress made, these methods do not explore local structures such as corners and often perform poorly when it comes to capturing detailed aspects of the point cloud.

Unlike the aforementioned methods, our PA-NET synchronizes the refinement of local structures while maintaining global performance by splitting the overall point cloud into several parts of points with similar geometric properties and optimizing these properties within each part. This approach explicitly guides the network to learn the representation of local features, resulting in superior performance in localized parts compared with the methods mentioned above.

### 2.2. Point Cloud Part Segmentation

Point cloud part segmentation aims to assign each point of an object to different part classes. Due to the challenges associated with labeling, existing point cloud part segmentation datasets are limited in size and primarily consist of ShapeNet-part [17] and PartNet [18]. Current methods in this domain can be categorized into supervised part segmentation and semi-supervised part segmentation. Kpconv [19] is a representative supervised method that intelligently selects *K* key points in the point cloud to efficiently aggregate features, achieving commendable performance. PartNet [20] also falls under the supervised category; it divides the point cloud according to a tree structure, resulting in robust and explainable part segmentation. Semi-supervised methods [21,22] employ techniques such as sampling key points and clustering to assist the network in achieving effective part segmentation. These methods bridge the gap between supervised and unsupervised learning, leveraging partial annotations for improved segmentation results.

While the studies mentioned above have made progress, their part definitions do not align with our method’s part requirements. To address this, we drew inspiration from Large Language Models (LLMs). We adopted a question-and-answer format to obtain annotated information tailored to our approach. Additionally, we designed multi-modal modules to acquire part-specific knowledge, enabling precise and controllable part segmentation.

### 2.3. Multi-Modal Method of Point Cloud

Multi-modal deep learning has emerged as a powerful approach to advancing point cloud processing tasks. By integrating complementary information from various sources such as images and texts, multi-modal networks can learn more distinctive representations, leading to significant improvements in performance. In the realm of visual modality, a common technique involves merging Li-DAR data with camera images to enhance 3D object detection [23]. Additionally, pioneering work by Su et al. [24] introduced a deep neural network that combines point clouds with texture images, resulting in improved semantic segmentation outcomes. These studies highlight the value of incorporating visual features from images to enhance point cloud analytics. In the realm of textual modality, innovations like Text2mesh [25] utilize both point clouds and textual descriptions for 3D model retrieval, generating more relevant and accurate results. Moreover, the advent of large models such as CLIP [26] has significantly contributed to the multi-modal evolution of point cloud applications. Recent works by Zhang et al. [27] and Huang et al. [28] employ novel image–text models to distillate 3D networks, further advancing the capabilities of multi-modal point cloud processing.

Indeed, while there have been efforts in the realm of multi-modal methods, applying them to part segmentation remains a challenging endeavor. The intricate and nuanced structures of components make it exceptionally difficult to transfer this knowledge effectively.

## 3. Method

### 3.1. Overview

**Figure 4:**
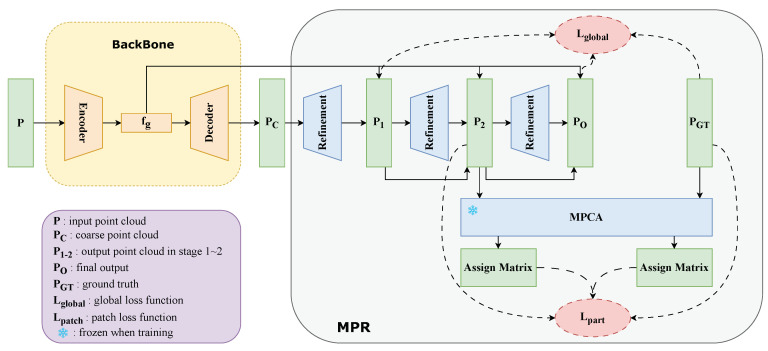
The framework of our method involves a plug-in module called MPR, which can be integrated after a standard backbone, enhancing the performance concerning local structures. The entire module comprises three refinement stages, with each stage utilizing a refinement network to enhance the quality of the point cloud. MPCA is employed to guide the network’s attention towards local structures and is added after the second stage. Within this framework, the loss function constrains the network at both global and local levels.

As illustrated in Figure 4, our network, PA-NET, mainly comprises two modules: a backbone network and a refinement framework named MPR.

#### 3.1.1. Backbone Network

We utilize a standard encoder–decoder framework as our backbone. Let $P=\{p_{i}\}$ represent the input point cloud, where 0≤i≤N. Here, *N* is the number of point clouds, and $p_{i}$ denotes a point in 3D space having a shape of 1×3. Employing three feature extraction layers from Snowflake [8] as the encoder, we transform the point features (N×3) into a global feature, $f_{g}$, with a shape of 1×512. The decoder’s objective is to generate a coarse point cloud, $P_{C}$, guided by global feature $f_{g}$. The structure of the decoder mirrors is that of Snowflake, enabling the generation of the coarse point cloud with dimensions 512×3.

#### 3.1.2. MPR Module

The MPR module, designed as a plug-in component, can be seamlessly integrated after various standard backbones, enhancing the quality of locally generated structures. This module consists of three stages, refining the generated point cloud incrementally. Denoting the shapes of the output point clouds as $P_{1}$, $P_{2}$ and $P_{O}$, with $N_{1}$, $N_{2}$ and $N_{O}$ points, respectively, the number of point clouds increases in each progressive stage. The entire module comprises two crucial sub-modules and their corresponding loss functions, which will be elaborated upon in the subsequent sections.

### 3.2. Refinement Sub-Module

**Figure 5:**
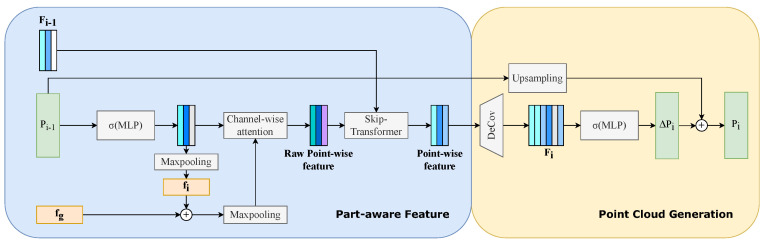
Refinement sub-module: This module comprises two essential components—part-aware feature extraction and point cloud generation.

As shown in Figure 5, the refinement module is designed to direct the network’s focus towards specific regions of the point cloud. It takes as input the point features output by i−1-th layer $F_{i-1}$, the coarse point cloud generated by i−1-th layers $P_{i-1}$ and global feature $f_{g}$. Within this module, a multi-layer perceptron (MLP) network extracts global feature $f_{i}$ from $P_{i-1}$. Subsequently, $f_{i}$ and $f_{g}$ are concatenated and passed through a max-pooling layer to serve as weights for channel-wise attention applied to the row-wise point features. Guided by this combined global feature, the attention of the model becomes more focused on local regions. To capture local context, we employ a skip-transformer [8], which takes $F_{i-1}$ and the row-wise point features as input. The skip-transformer facilitates step-by-step optimization in all three refinement stages, enhancing the point-wise features in each stage. Using these features, a more detailed point cloud is generated through shift prediction and upsampling. A de-convolution layer is employed for upsampling the point-wise features to match the desired output shape. Three MLP layers predict the shift ($\Delta P_{i}$) for each point, with $F_{i}$ as input. The $P_{i-1}$ coarse point cloud is upsampled to twice its size, and the shifts are applied to produce the final output point cloud in the *i*-th layer.

### 3.3. MPCA Module

#### 3.3.1. Textual Embedding Generation

Inspired by [29], we developed a Q&A-based method to acquire information about parts, addressing the challenge of obtaining relevant prior knowledge due to the limitations of existing part segmentation datasets in meeting our specific requirements for part segmentation.

For a given point cloud *P*, we captured multiple photos from various angles by rendering the point cloud and taking images using Cloud Compare software (https://www.cloudcompare.org/). For each point cloud, we initially positioned its center of gravity at the coordinate origin. Subsequently, we placed the camera at the appropriate location on its side. We then rotated the point cloud and captured an image every 30°, resulting in a total of 12 images taken from various perspectives.

These photos served as queries in conjunction with a series of part-related language prompts. For each category in the dataset, we manually labeled the potential parts it might have contained. For instance, a chair could include parts such as “Leg”, “Armrest”, “Seat”, “Backrest” and “Footrest”. For each component, we created two types of questions: an existential one and a quantitative one. The existential question was formulated as “Does the [CLASS] have a [PART]?”, and the quantitative one, as “How many [PART] does the [CLASS] have?” Here, [CLASS] represents the category of the object obtained from the ground truth, and [PART] is a pre-defined component. When a part of an object does not exist, no questions about the quantity are asked.

**Figure 6:**
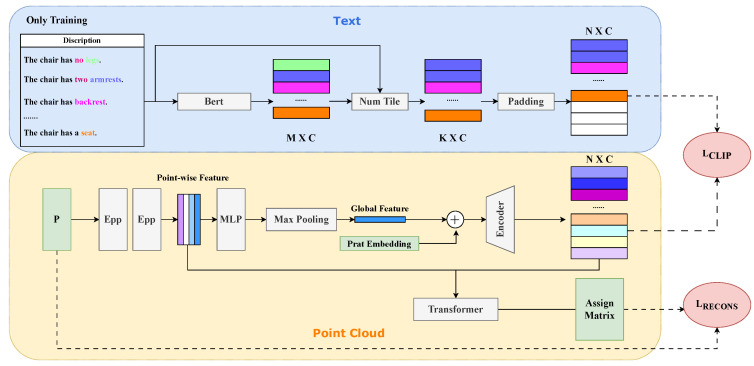
MPCA module: This module is trained using a multi-modal approach. Once the training is complete, the parameters are frozen and integrated into the backbone framework. This integration allows the module to split the point cloud into local parts.

Using this template, we swiftly obtained text labels for the parts of a class of objects. For each object, we amalgamated the answers to the two questions and inserted them into the template “The [CLASS] has [NUM] [PART]”, where [NUM] signifies the number of corresponding parts. This process resulted in a list of parts associated with each object, where each list item described a specific part of the object, as illustrated in Figure 6.

Subsequently, we utilized a pre-trained Bert [26] to project these sentences into a textual embedding space. Once we obtained textual embeddings with dimensions M×C, we replicated embedding K×C based on the number of distinct parts using a Num Tile module, which can tile the embeddings with the number of the corresponding part. Here, *N* represents the maximum number of parts a single object can be divided into. If K≤N, the padding module added blank vectors until K=N was achieved. The resulting textual embedding, N×C, represented the part features of the object’s segmentation.

#### 3.3.2. Framework

The entire framework takes a point cloud as input. Initially, two EdgeConv (Egg) layers [30] are employed to generate a 64D point-wise feature for each point. These features are then passed through MLP layers and a max-pooling layer to obtain a 512D global feature. A part embedding matrix of dimensions N×512 is created to be concatenated with the global feature, resulting in the initial part features. This part embedding is essentially a one-hot matrix encoding the part order.

Subsequently, the combined features, totaling 2N×512, are fed into an encoder to calculate the part features, resulting in a matrix of dimensions N×C. A loss function measures the distance between the output part features and the textual embedding. To obtain the assignment matrix, the point-wise features and part features are used as input for a transformer, which calculates the relationships between these features. The final assignment matrix (N×M) is constrained with reconstruction loss to ensure the accuracy of the assignment matrix, decoded by the transformer layer. This process ensures the effective alignment of part features and textual embeddings.

#### 3.3.3. Loss Function of MPCA

The loss function mainly contains two parts, CLIP loss and reconstruction loss. CLIP loss is inspired by CLIP [26], aligning features between modalities effectively. Reconstruction loss is used to force the points assigned to the same part to form the same local structure.

➢**CLIP loss.** The CLIP loss function serves two primary purposes: firstly, it efficiently aligns features between modalities, and secondly, it guides the network to encode part features in a specific order. The application of this loss function ensures that the split point clouds generated by the network carry specific semantic meanings.

The CLIP loss function is defined by Formula (1).

(1) $$L_{C}=\frac{1}{2}\left[\frac{1}{B}\sum_{k=1}^{B}\frac{\exp\left(S_{v_{k},t_{k}}/\tau \right)}{\sum_{l}^{B}\exp\left(S_{v_{k},t_{l}}/\tau \right)}+\frac{1}{B}\sum_{k=1}^{B}\frac{\exp\left(S_{v_{k},t_{k}}/\tau \right)}{\sum_{l}^{B}\exp\left(S_{v_{l},t_{k}}/\tau \right)}\right],$$

where *B* is the batch size and τ is the temperature hyperparameter. $S_{v,t}$ is the total similarity score, defined by Formula (2):

(2) $$S_{v,t}=\frac{1}{2}\left(\sum_{i=1}^{N_{v}}w_{v}^{i}\max_{j}a_{ij}+\sum_{j=1}^{N_{t}}w_{t}^{i}\max_{i}a_{ij}\right).$$

Variables *u* and *v* represent two different modalities, such as text and point clouds, while $a_{ij}$ is a feature similarity matrix obtained by multiplying features from different modalities. The weights of the modal features are represented by $w_{v}^{i}$ and $w_{t}^{j}$, obtained as $\left[w_{v}^{0},w_{v}^{1},...,w_{v}^{N_{v}}\right]=\mathrm{Softmax}\left({\mathrm{MLP}}_{v}\left(V_{f}\right)\right)$, where ${\mathrm{MLP}}_{v}$ represents the fully connected layers used to encode modal *v*.

➢**Reconstruction loss.** To efficiently guide the point cloud assignment results, we employ the reconstruction loss to constrain the point cloud assignment process. This approach, commonly used in unsupervised part segmentation, assumes that each part can be enclosed by a minimum rectangle. We follow the method used in CAVS [31] for reconstruction, utilizing the point cloud assignment matrix output by MPCA.

The reconstruction loss is defined by Formula (3).

(3) $$L_{rec}=\frac{1}{N}\sum_{n=1}^{N}\sum_{m=1}^{M}W_{m,n}d\left(p_{n},part_{m}\right),$$

where $W_{m,n}$ represent the probability of the *n*-th point belong to the *m*-th part; *W* is the assignment matrix calculated by the network; d(·,·) represents the distance between a point and the part, reconstructed using the method from CAVS [31]. We apply the same method to calculate distances as in CAVS [31].

### 3.4. Loss Function

The loss function guides the network to learn the generation of the complete point cloud both globally and locally. The global loss function ($L_{global}$) employs the common CD loss, which ensures that the generated point cloud closely matches the ground truth globally. The local loss function ($L_{part}$) measures the distance between the generated result and the ground truth for each corresponding part, directing the network’s attention to local details. The overall loss function is defined by Formula (4), where α and β represent the weights of each loss function. The specific definitions of each loss function are provided below.

(4) $$L=\alpha L_{global}+\beta L_{part}.$$

#### 3.4.1. Global Loss


(5) $$L_{global}=\frac{1}{P}\sum_{x\in P}\min_{y\in Q}||x-{y||}^{2}+\frac{1}{Q}\sum_{y\in Q}\min_{x\in P}||y-{x||}^{2}.$$

The global loss function ($L_{global}$) is defined by Formula (5), where *P* and *Q* represent the generated point cloud and ground truth, respectively. *x* and *y* denote individual points in point clouds *P* and *Q*. This formula ensures that the two point clouds are as close as possible globally.

#### 3.4.2. Part Loss

The part loss ($L_{part}$) constrains the distance between points in the corresponding parts of the generated point cloud and the ground truth, directing the network’s attention to local parts. This is achieved using the point-to-part assignment matrix output by MPCA. The loss function is defined bidirectionally, as shown in Formula (6), where *P* and *Q* represent the generated point cloud and ground truth, respectively, and *M* and *N* represent the assignment matrices corresponding to *P* and *Q*.

(6) $$L_{part}=D_{part}\left(P,Q,M,N\right)+D_{part}\left(Q,P,N,M\right).$$

$D_{part}$ is defined as the distance function between two point clouds guided by the assignment matrix, as shown in Formula (7). $D_{part}$ is a one-way distance metric, where *N* represents the number of points in point cloud *P*. In the formula, *k* represents the *k*-th part in the point cloud, and *m* is the maximum number of parts in one object.

(7) $$D_{part}\left(P,Q,M,N\right)=\frac{1}{N}\sum_{i=0}^{N}\min_{q_{j}\in Q,0\leq k\leq m}WD\left(p_{i},q_{j},M_{ik},N_{jk}\right).$$

In Formula (7), WD(·,·,·,·) is the weighted distance between two points, as defined by Formula (8). The weighted distance between points *p* and *q* is calculated as a weighted L2 distance, where the weight is determined by assignment probabilities *m* and *n*.

(8) $$WD\left(p,q,m,n\right)=\left(2-m\cdot n\right)\cdot ||p-{q||}^{2}.$$

The part loss quantifies the similarity between the generated point cloud and the ground truth at the local level. Its application directs the network’s focus, guiding the generation of more precise local structures.

## 4. Experiment

This section systematically evaluates our proposed method with a series of extensive experiments. We first present validation experiments to assess the efficacy of the MPCA sub-module, followed by a demonstration of the overall performance of the entire network in point cloud completion.

### 4.1. Experiment on MPCA Module

#### 4.1.1. Dataset and Settings

##### Implementation Details

Our experiments were conducted using an Nvidia GeForce GTX TITAN X graphics card with 12 GB of RAM. The training process employed the Adam optimizer with an exponential decay rate of 0.9 and an initial learning rate of $6\times 10^{-3}$. The data batch size was set to 32, and a total of 300 iterations were performed. Initially, the network was trained to align the features of the point cloud parts and textual embeddings for the first 100 iterations. Subsequently, the entire network was trained together for the remaining iterations.

##### Dataset

We trained the network using the ShapeNet-Part dataset [32], which consists of 16,881 shapes from 16 different categories with a total of 50 labeled parts. One of the main challenges posed by this dataset is the high imbalance among categories. Each shape in the dataset is sampled with 2048 points, and most shapes have fewer than six parts. It is important to note that we did not utilize the labels during training, reserving them only for evaluation purposes. The dataset was divided into a training set and a test set in an 8:2 ratio.

#### 4.1.2. Visualization Results on ShapeNet-Part

**Figure 7:**
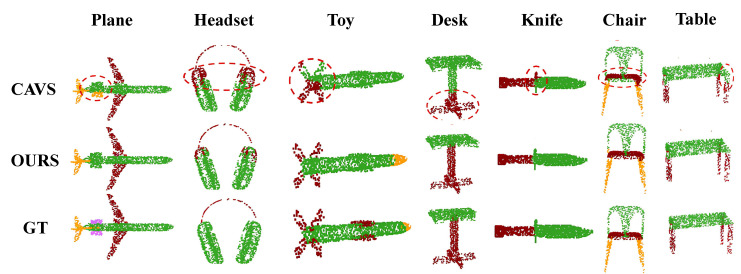
The visualization results on ShapeNet-Part [32] are presented in the figure, with a comparison made using the novel unsupervised method CAVS [31]. The distinctive advantages of our approach are particularly evident in the portion of the figure encircled by the red line.

To showcase the effectiveness of our approach, we assigned each point cloud to the part with the highest probability and visualized the results by assigning distinct colors to different parts. This visualization was conducted on the test set, and we chose the novel unsupervised method CAVS [31] for comparison. We selected seven categories that are commonly encountered in point cloud completion for this comparison, as depicted in Figure 7.

The results clearly demonstrate the efficacy of our method in accurately dividing point clouds into several geometrically similar parts. Notably, the circled area in the figure highlights instances where our method outperforms CAVS. Furthermore, our approach consistently produces reasonable results across various categories, showcasing its ability to accurately segment point clouds into parts suitable for point cloud completion.

#### 4.1.3. Visualization Result on Completion3D Dataset

**Figure 8:**
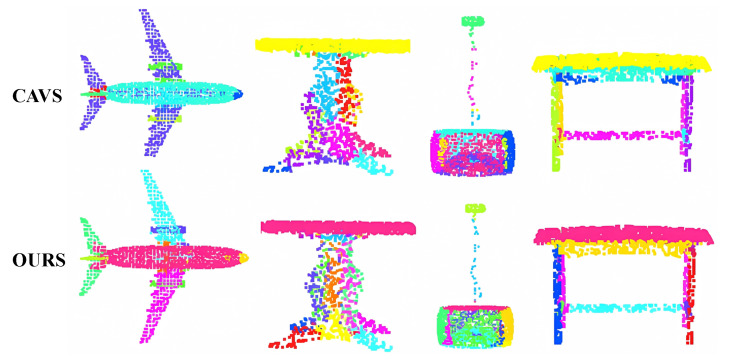
Visualization results on Completion3D dataset. The figure demonstrates the capability of our method to effectively divide the point cloud into reasonable parts.

In our experiments on the Completion3D dataset, we validated the effectiveness of our method as a point assignment module. Throughout the completion process, we extracted the assignment matrix of the point cloud and utilized the visualization method outlined in Section 4.1.2. The results are displayed in Figure 8. Notably, CAVS [31] exhibits highly unfavorable assignment outcomes on previously unseen data. In contrast, our method, trained using multi-modal information, demonstrates robustness on new data and effectively divides the point cloud into logical parts. The incorporation of MPCA can divide the point cloud into meaningful parts, leading to these positive outcomes.

### 4.2. Experiment on Point Cloud Completion

#### 4.2.1. Dataset and Settings

##### Implementation Details

In our experiments, we utilized a GeForce RTX 3090 graphics card with 24 GB of RAM. The training process employed the Adam optimizer with an exponential decay rate of 0.9 and an initial learning rate of 0.001. The learning rate was halved every 50 rounds, and a data batch size of 32 was set, totaling 150 iterations. During training, we set hyperparameters α and β of the loss function to 0.5 each, ensuring equal influence from both loss functions on the network.

##### Dataset

To comprehensively evaluate the effectiveness of our method, we selected two widely used benchmarks, PCN [9] and Completion3D [33]:

- The PCN [9] dataset consists of eight categories. Incomplete shapes are generated by back-projecting complete shapes into eight different partial views. We followed the settings of Snowflake [8] to align the ground truth.
- The Completion3D dataset [33] comprises a total of 30,974 objects across eight categories, with each object containing an incomplete point cloud and its corresponding ground truth. Each complete point cloud consists of 2048 points, while the incomplete point cloud is obtained by back-projecting depth images into 3D space, resulting in a varying number of points. We divided the dataset into training, validation and test sets following the Completion3D protocol.

##### Evaluation Metric

We employed the widely used evaluation metric, Chamfer Distance (CD), for our evaluations. Consistent with established practices, we utilized the $L_{1}$ version of CD for the PCN dataset and the $L_{2}$ version of CD for the Completion3D dataset, adhering to the settings of previous methods.

#### 4.2.2. Quantitative Analysis

##### Experiments on PCN Dataset

Table 1 presents the quantitative results of our method in comparison to other methods on the PCN dataset [9]. We meticulously reproduced these methods locally and conducted the experiments. Clearly, our method achieves novel performance, with an average Chamfer Distance value of 7.24 compared with 7.32 in the other methods. Particularly, for categories with well-defined local structures, such as Chair and Table, our method outperforms other methods significantly. We attribute this success to our method’s ability to effectively segment these parts and optimize the local structure, leading to superior outcomes.

However, there are categories, such as Plane and Watercraft, where our results are slightly inferior. This discrepancy might arise from the substantial individual differences within these categories and their unclear structures. Additionally, the accuracy is notably influenced by the composition of the test set, potentially introducing errors into the results.

In summary, our method excels on the PCN dataset, demonstrating superior performance in terms of average Chamfer Distance across most categories. This showcases the capability of our approach.

##### Experiments on Completion3D Dataset

In Table 2, we present the quantitative results of our method alongside other methods on the Completion3D dataset [33]. We meticulously replicated these methods locally and conducted the experiments. The table clearly indicates that our method achieves novel performance, with an average Chamfer Distance value of 8.13, surpassing the other methods, which score 8.37. Our method achieves much more accuracy than Snowflake [8] in most categories, especially in Table (12.32 vs. 13.93).

Upon observation, our method demonstrates superior accuracy in categories with distinct local structures, such as Cabinet, Chair and Table. However, for categories like Plane, Car and Couch, our accuracy is comparable to Snowflake’s.

In summary, the results on the Completion3D dataset underline our method’s ability to generate more accurate local structures from incomplete point clouds.

##### Ablation Study

➢**Point cloud assignment methods.** To assess the effectiveness of our MPCA module, we conducted ablation experiments on this component. We replaced the MPCA module with a clustering method employing different numbers of clusters (denoted by *K*) for predicting the point cloud assignment matrix. Specifically, we tested *K* values of 1, 2, 4, 8 and 16 in our experiments. For each setting, we computed clusters for both the point cloud output from the network and the ground truth, aligning the split parts by calculating the distance between the cluster centers.

**Figure 9:**
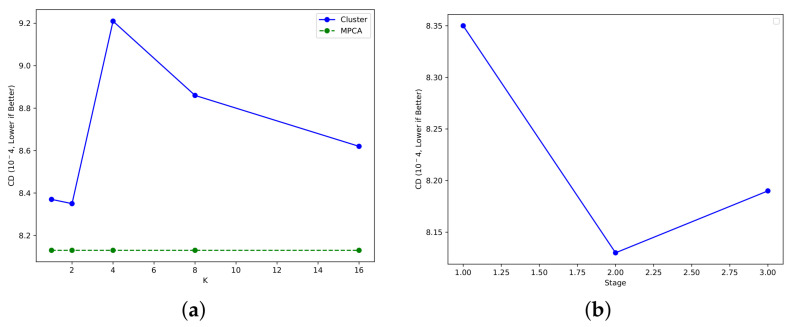
Results of ablation study with point cloud assignment methods (**a**) and part loss (**b**). Per-point $L_{2}$ Chamfer Distance $\,10^{-4}$ is used as matrix. (Lower is better).

The experimental results, presented in Figure 9a, demonstrate the average Chamfer Distance under various configurations, allowing us to evaluate performance with different settings. As depicted in the table, when K=1, it signifies that no parts are split for the point cloud. Using a simple clustering approach to divide the point cloud results in a slight improvement in accuracy with fewer divisions (K=2). However, as the number of clustering centers increases (K=4, K=8), it leads to instability in the completion effect and a decrease in accuracy. We attribute this to significant differences in the distribution between the network output and the ground-truth point cloud, causing variations in the clustering effect.

This ablation study underscores the robustness of our method in achieving assignment results. It effectively transforms global optimization into local optimization, guiding the network to perform better in terms of accuracy and stability.

➢**Loss Function.** We initially conducted ablation experiments on the existential aspect of our loss function. This involved replacing all the loss functions of the three stages with a specific loss function, and the resultant outcomes are presented in Table 3. The table clearly indicates that employing any of the loss functions in isolation does not yield optimal results. This substantiates the necessity of both global optimization and local optimization to complement each other for achieving the best outcomes.

We also explored the impact of hyperparameters α and β. Our empirical findings demonstrate that larger α, indicating a greater weight for the global loss, results in better performance for categories with local ill-structured shapes, such as Car. Conversely, larger β leads to improved performance in categories with more well-defined local structures, such as Chair. To enhance the network’s generalization capability, we set the values of α and β to 0.5 when computing the final result, ensuring their equal influence on the network.

In addition, as shown in our framework, the refinement process for coarse point clouds is divided into three stages, with our part loss function being applied to the output of the second stage. To understand whether imposing this constraint yields different effects in different stages, we conducted an ablation experiment. We denoted the stage in which the part loss function is imposed as *p*. The results of this ablation study are presented in Figure 9b.

The results show that the network’s performance improves whenever the loss function is imposed. However, introducing the loss function too early (p=1) causes its effect to be overshadowed by the CD loss. On the other hand, incorporating the loss function in the final stage amplifies the effects of noise, leading to inferior results. Therefore, integrating the part loss function in the intermediate stage yields the most significant enhancement.

##### Plug-in Experiment

MPR serves as a plug-in framework. To demonstrate its ability to enhance the accuracy of the baseline, we conducted a simple experiment. We selected PCN and Snowflake as the baseline networks and integrated MPR into them. Both networks were tested on Completion3D, and the quantitative results are presented in Table 4.

The results are evident: when our MPR module is added to PCN, the accuracy significantly improves (15.32 vs. 17.80). Similar enhancements were observed in our experiments using Snowflake [8] as the backbone network (8.13 vs. 8.37).

#### 4.2.3. Visualization

##### Visualization Results on Completion3D

**Figure 10:**
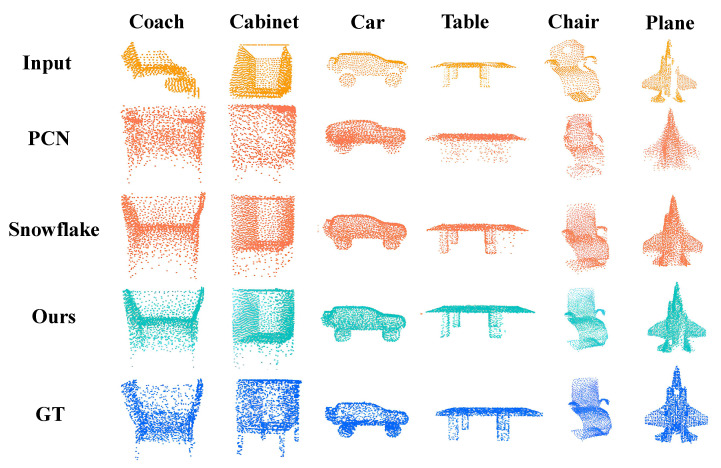
Visualization results. In our comparative visualization with the classical method PCN [9] and the state-of-the-art method, Snowflake [8], significant differences in the generated results are evident. Our method excels at producing finer details, outperforming Snowflake [8], particularly at object edges and in localized areas.

We conducted experiments on the Completion3D dataset [33]. For visualization, we selected the classical method PCN [9], as well as the current state-of-the-art method, Snowflake [8]. The visualization results are depicted in Figure 10. In the figure, we present the point clouds of input, output from PCN, output from Snowflake, output from our network, and ground truth separately. We chose six common types of objects to showcase the results.

It is evident from the visualization that our method significantly outperforms PCN. While Snowflake achieves good results, there are still outline points visible at the edges, as observed in the cabinet and table examples. Additionally, their method does not provide a clear local structure, as seen in the plane example. In contrast, our method demonstrates a substantial improvement over PCN and excels at optimizing local details compared with Snowflake.

##### Visualization Result on Chair Category

**Figure 11:**
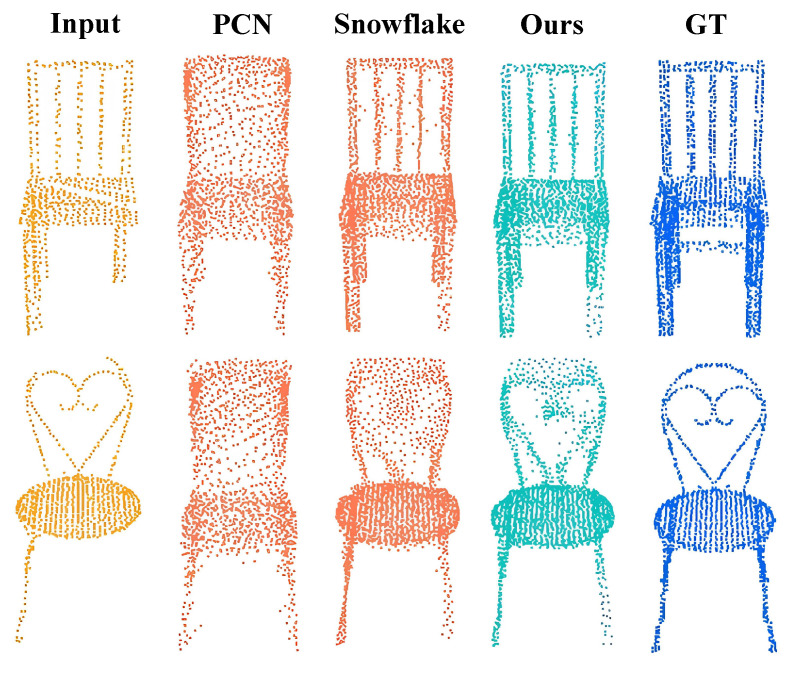
Visualization results on Chair. We are confident that our method excels at generating precise local structures, especially for objects with distinct and intricate local features. To underscore this capability, we provide additional visualizations of completion results for chairs with complex local structures, comparing our approach with PCN [9] and Snowflake [8]. These visualizations distinctly showcase our method’s ability to produce highly accurate local structures.

From the findings presented in Section 4.2.2, our approach, which involves global–local co-optimization through part division, significantly enhances completion performance in categories with distinct and complex structures, such as Chair and Desk. To further illustrate this, we provide visualization results specifically focusing on Chair, a category with intricate local structures. As depicted in Figure 11, we compared our method with PCN [9] and Snowflake [8]. The comparison vividly demonstrates that our method excels at preserving local details with remarkable clarity in comparison to the other techniques. This highlights our method’s exceptional ability to generate intricate and finely detailed local structures.

## 5. Conclusions

Point cloud completion plays a significant role as an upstream task in 3D vision. However, many existing point cloud completion methods suffer from unclear local structures. In this paper, we analyze the limitations of Chamfer Distance and propose a part-aware point cloud completion method that avoids falling into local optima by transforming global optimization into a local–global co-optimization. Firstly, to achieve robust point-to-part assignment, we introduce a multi-modal point cloud assignment (MPCA) module that can split the coarse point cloud and ground truth into parts in a specific order. Then, using this module, we present a novel completion framework designed to focus the network’s attention on local structures. Importantly, our proposed module, MPR, is a plug-in module. The generation of fine-grained local structures can be enhanced by adding this module after different backbones. We conducted extensive experiments to validate our method. The experimental results demonstrate that our approach achieves novel performance in accuracy. Visualization results further illustrate that our method excels at generating intricate local structures.

## Figures and Tables

**Table 1:** Point cloud completion on PCN in terms of per-point $L_{1}$ Chamfer Distance $\,10^{-3}$ (lower is better).

Method	Plane	Cabinet	Car	Chair	Lamp	Couch	Table	Watercraft	Average
PCN [9]	6.03		21.67	11.12	8.73	10.68	11.53	12.04	10.23
TopNet [33]	7.26	13.26	10.68	14.24	14.69	14.55	11.67	10.98	12.16
GRNet [34]	6.23	10.29	9.65	9.33	8.16	10.46	8.37	8.22	8.84
Snowflake [8]	**4.35 **	9.32	8.52	7.51	6.21	**9.42**	6.42	**6.78**	7.32
Ours	4.41	**9.14**	**8.52**	**7.09**	**6.19**	9.65	**6.13**	6.82	**7.24**

**Table 2:** Point cloud completion on Completion3D in terms of per-point $L_{2}$ Chamfer Distance $\,10^{-4}$ (lower is better).

Method	Plane	Cabinet	Car	Chair	Lamp	Couch	Table	Watercraft	Average
PCN [9]	9.68	21.04	12.87	24.76	22.14	20.02	19.93	11.92	17.80
TopNet [33]	7.29	18.38	12.69	19.34	14.28	16.12	14.78	8.86	13.96
SA-Net [1]	4.29	12.41	6.79	11.64	11.73	12.13	11.75	7.89	9.83
GRNet [34]	5.83	15.62	7.32	10.32	10.05	9.39	12.02	6.03	9.57
VE-PCN [35]	2.56	12.03	6.23	10.04	9.62	9.10	15.10	4.72	8.68
Snowflake [8]	**2.08 **	11.55	**5.94**	10.48	9.71	**8.40**	13.93	4.90	8.37
Ours	2.25	**11.35**	6.08	**10.06**	**9.52**	8.70	**12.32**	**4.71**	**8.13**

**Table 3:** Ablation study on loss function on the Completion3D in terms of per-point $L_{2}$ Chamfer Distance $\,10^{-4}$ (lower is better).

Global Loss	Part Loss	Airplane	Cabinet	Car	Chair	Lamp	Couch	Table	Watercraft	Average
✗	✓	**2.07**	11.6	6.17	10.54	9.85	9.38	12.66	**4.63**	8.39
✓	✗	2.08	11.55	**5.94**	10.48	9.71	**8.40**	13.93	4.90	8.37
✓	✓	2.25	**11.35**	6.08	**10.06**	**9.52**	8.70	**12.32**	4.71	**8.13**

**Table 4:** Plug-in experiment on the Completion3D on terms of per-point $L_{2}$ Chamfer Distance $\,10^{-4}$ (lower is better).

Method	Plane	Cabinet	Car	Chair	Lamp	Couch	Table	Watercraft	Average
PCN [9]	9.68	21.04	12.87	24.76	22.14	20.02	19.93	11.92	17.80
PCN + MPR	**8.32**	**16.03**	**11.06**	**22.04**	**18.32**	17.63	**16.67**	**10.96**	**15.32**
Snowflake [8]	**2.08**	11.55	**5.94**	10.48	9.71	**8.40**	13.93	4.90	8.37
Snowflake + MPR	2.25	**11.35**	6.08	**10.06**	**9.52**	8.70	**12.32**	**4.71**	**8.13**

## Data Availability

No new data were created or analyzed in this study. Data sharing is not applicable to this article.
